# Case report: Hypnic headache responds to agomelatine–a potential prophylactic treatment option

**DOI:** 10.3389/fneur.2023.1179391

**Published:** 2023-06-23

**Authors:** Sui-yi Xu, Ling Li, Wen-xiu Sun, Jia-yu Shen, Chang-xin Li

**Affiliations:** Department of Neurology, Headache Center, The First Hospital of Shanxi Medical University, Taiyuan, Shanxi, China

**Keywords:** Hypnic headache, circadian rhythm, agomelatine, melatonin, serotonin

## Abstract

**Introduction:**

Hypnic headache (HH) is a rare primary headache that is characterized by strict sleep-related attacks. However, the pathophysiology of HH remains unclear. The nocturnal nature of this activity suggests a hypothalamic involvement. The pathogenesis of HH may involve the brain structure that regulates circadian rhythms and is related to an imbalance between hormones, such as melatonin and serotonin. Currently, evidence-based medicine for HH pharmacotherapy is lacking. Acute and prophylactic treatment of HH is based on only a few case reports. Here, we report a case study in which agomelatine showed desirable responsiveness for the prophylactic treatment of HH for the first time.

**Case description:**

We present the case of a 58-year-old woman with a 3-year history of nocturnal left temporal pain that awakened her during the wee hours. Brain magnetic resonance imaging did not reveal any midline structural abnormalities associated with circadian rhythms. Polysomnography revealed headache-related awakening at approximately 5:40 am, after the last rapid eye movement phase. No sleep apnea-hypopnea events were observed, without oxygen saturation or blood pressure abnormalities. The patient was prescribed agomelatine 25 mg at bedtime as a prophylactic treatment. In the following month, the frequency and severity of the headaches decreased by 80%. After 3 months, the patient’s headache completely resolved, and the medication was discontinued.

**Conclusion:**

HH only occurs during sleep in the real world, leading to substantial sleep disturbances in older populations. Headache center neurologists need to focus on the prophylactic treatment of patients before bedtime to avoid nocturnal awakening. Agomelatine is a potential prophylactic treatment option for patients with HH.

## Introduction

Hypnic headache (HH) is a rare primary headache that was first described by Raskin in 1988 (1) and is characterized by strict sleep-related headache attacks. HH was once named clockwise or alarm clock headache because it only occurs during sleep and almost always at the same time (2). The International Headache Society suggests that the following criteria must be met for the headache to be diagnosed as HH (3): (1) it develops only during sleep and causes awakening, (2) it occurs for 10 days/month for >3 months, (3) it lasts from 15 min up to 4 h after awakening, and (4) there are no cranial autonomic symptoms or restlessness, according to the International Classification of Headache Disorders, 3rd edition (ICHD-3). The diagnosis of HH requires the exclusion of secondary causes of nocturnal headaches, such as nocturnal hypertension (4), hypoglycemia (5), auditory neuroma (6), and influenza A virus infection (7). Cervicogenic headaches can also awaken older adults from sleep. Headache may be caused by abnormal neck posture during sleep or degenerative cervical spine disease that compresses nerve roots (8). Obstructive sleep apnea syndrome (OSAS) is a common cause of nocturnal awakening and may be involved in HH (9). However, the presence of an OSAS does not necessarily exclude the possibility of HH (3).

HH is more frequent after the age of 50 years and is more common in women but may also occur in young adults and children (10). It usually occurs in the whole brain but may also occur bilaterally in the frontotemporal lobe and rarely in the occipital lobe as moderate to severe dull pain (11). However, epidemiological data regarding HH are limited. A recent Icelandic study suggested that the prevalence of HH is 0.22% (12). However, the pathophysiology of HH remains unclear. Previously, HH episodes were thought to occur strictly during rapid eye movement (REM) (13). However, subsequent reports have shown that many HHs occur during non-REM (14). The nocturnal nature of this activity suggests hypothalamic involvement (11, 15). The hypothalamus is considered a major integration center that regulates the neurological and endocrine systems. It has a reciprocal influence on pain control and sleep regulation owing to its close connection with the periaqueductal gray matter, locus coeruleus, and median raphe nucleus (16). The suprachiasmatic nucleus (SCN) in the anterior hypothalamus is the internal clock that regulates the sleep–wake state and is governed by the circadian cycle (17). With age, the hypothalamic-pineal axis, especially the SCN, becomes less functional, resulting in reduced melatonin secretion (18). This hypothesis is supported by the significant reduction in posterior hypothalamic gray matter volume in a voxel-based morphometric (VBM) study of patients with HH (15). Recently, an Indian study confirmed the loss of posterior hypothalamic gray matter using VBM-magnetic resonance imaging (MRI) (19). Moreover, fluctuations in melatonin levels alone do not seem to explain HH pathogenesis. Serum melatonin levels usually peak between 2:00 am and 5:00 am. In a clinical study, non-fasting serum was extracted at five different time points (12 noon, 4 pm, 7 pm, 10 pm, and 8 am). No significant changes in melatonin levels were detected between patients with HH and healthy controls (20). In summary, the pathogenesis of HH may involve the brain structure that regulates circadian rhythms and is related to an imbalance between hormones, such as melatonin and serotonin (10). Agomelatine is a selective melatonin receptor agonist (MT1/MT2) and a serotonin receptor antagonist (5-HT2C). In addition to its antidepressant effects, agomelatine is involved in the resynchronization of interrupted circadian rhythms, with beneficial effects on sleep architectures (21). Here, we report a case study in which agomelatine showed desirable responsiveness for the prophylactic treatment of HH for the first time.

## Case description

We present the case of a 58-year-old woman with a 3-year history of nocturnal left temporal pain that awakened her during the wee hours. Headaches were dull and not accompanied by migraine-related symptoms, such as nausea, vomiting, photophobia, phonophobia, blurred vision, flashing light, amaurosis, diplopia, tinnitus, and numbness or weakness of the body limbs. There were no cranial autonomic symptoms, such as conjunctival congestion, tearing, nasal congestion, runny nose, sweating, or ptosis, which are helpful in distinguishing between the types or subtypes of trigeminal autonomic cephalalgias, especially cluster headache. The headache lasted for approximately 2 h, even after the patient was awake, and the frequency of attacks was >15 days per month. The patient did not snore and denied a family history of headaches. The patient had a medical history of asthma and gastric ulcer.

**Figure 1:**
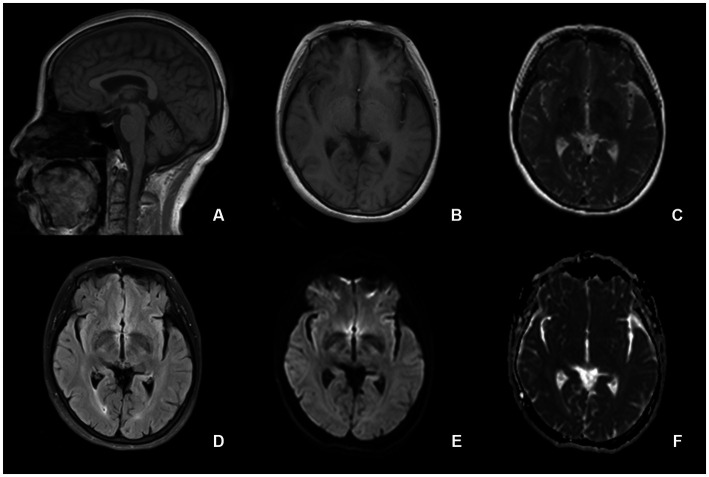
Brain magetic resonance imaging suggests normal structures. There are no midline structural abnormalities associated with circadian rhythms. **(A)** sagittal T1-weighted image, **(B)** axial T1-weighted image, **(C)** T2-weighted image, **(D)** fluid-attenuated inversion recovery, **(E)** diffusion-weighted imaging, and **(F)** apparent diffusion coefficient.

**Figure 2:**
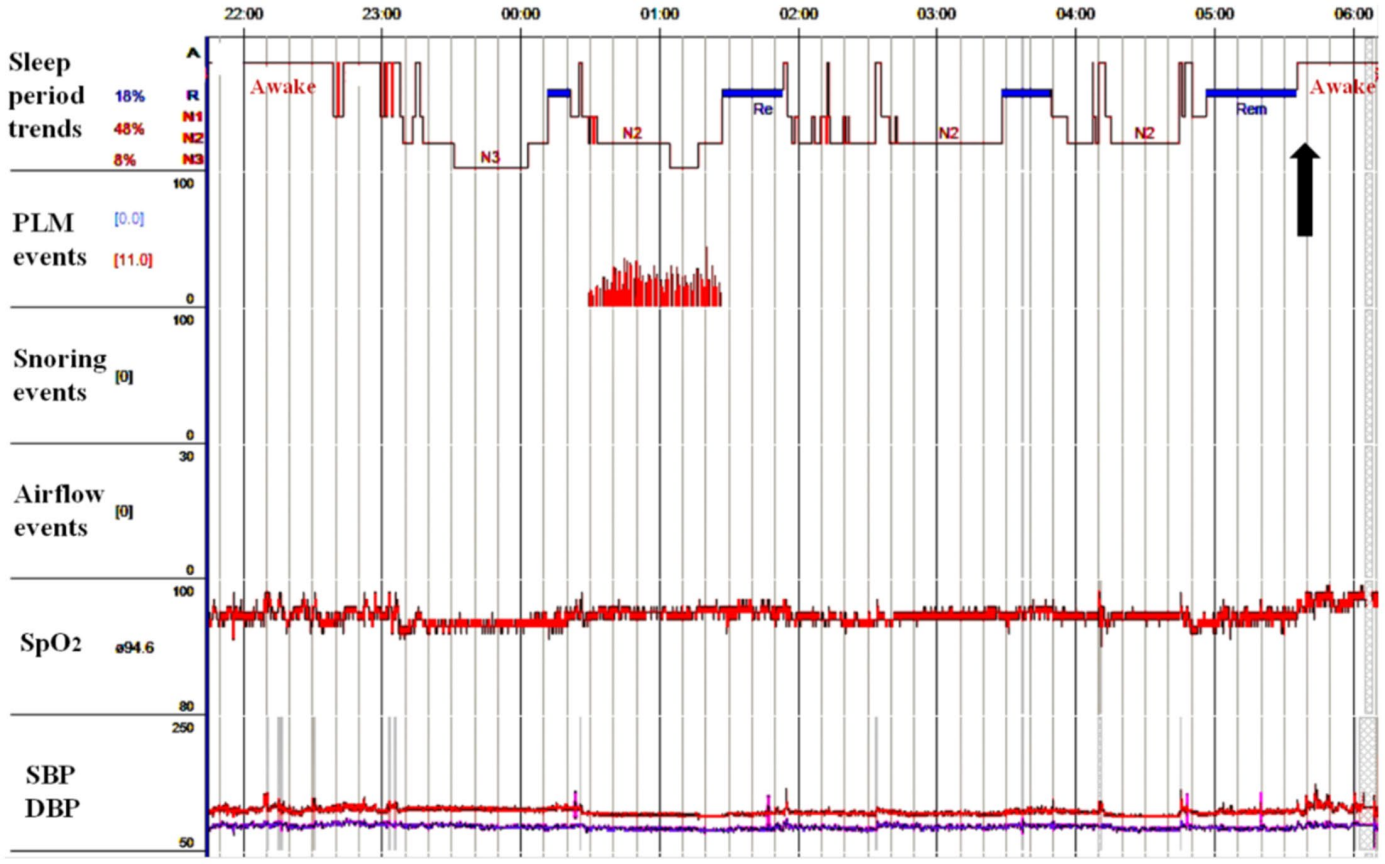
The sleep structure was approximately normal, with an increased proportion of N1 stage, a normal proportion of N2 stage, a decreased proportion of N3 stage, and a normal proportion of REM stage. Patient sat up with headache after the last REM sleep period at approximately 5:40 am (black arrow) without sleep apnea-hypopnea event observed. Meanwhile, the patient’s oxygen saturation and blood pressure were normal. PLM, periodic limb movement; SpO_2_, saturation of pulse oxygen; SBP, systolic blood pressure; DBP, diastolic blood pressure; REM, rapid eye movement.

On admission, her blood pressure was normal. No signs of nervous system impairment were observed. Serological analysis results, including hematology, electrolytes, fasting blood sugar, glycosylated hemoglobin, erythrocyte sedimentation rate, hepatic function, and renal function, were within normal limits. Brain MRI did not reveal any midline structural abnormalities associated with circadian rhythms (Figure 1). To explore the possible causes of secondary headaches, such as sleep apnea, nocturnal hypertension (22), and awakening from sleep, we performed polysomnography and ambulatory blood pressure monitoring (Figure 2). The patient was awakened by a headache at approximately 5:40 am after the last REM phase. No sleep apnea-hypopnea events were observed. The oxygen saturation and blood pressure were normal. The patient was diagnosed with HH according to the ICHD-3 guidelines and was prescribed agomelatine 25 mg at bedtime as a prophylactic treatment. In the following month, the frequency and severity of headaches decreased by 80%. After 3 months, the patient’s headache completely resolved, and the medication was discontinued.

## Discussion

Currently, evidence-based medicine for HH pharmacotherapy is lacking. Acute and prophylactic treatment of HH is based on only a few case reports. Caffeine, lithium, and indomethacin are the three most reported effective drugs for HH (8, 10, 11, 23, 24). A cup of strong coffee before bedtime appears to be a safe option for acute treatment. Caffeine is an antagonist of adenosine A1, A2A, and A2B receptors, and the cerebral A2 receptor is a key signaling molecule for sleep induction (25). In addition to drinking coffee at bedtime for HH prophylaxis, its use at night when waking up from pain can also shorten the duration and intensity of headaches (8). However, many people hesitate to use replacement therapies for coffee-induced insomnia. Most older individuals in Asia are not accustomed to drinking coffee (24). Lithium was the first drug reported for the prophylactic treatment of HH (1). Lithium regulates the biological clock by increasing the expression of the transcription factor BMAL1, which may help restore healthy circadian rhythms in patients (26). In addition, lithium may interact with NO and N-methyl-D-aspartate receptor signaling in the brain’s injury processing system (27). Lithium also downregulates serotonin receptors and increases serotonin release and transmission in the central nervous system (28) while indirectly increasing serum melatonin levels (29). A case series reported that lithium treatment was effective in >70.0% of the patients (30). A dose of 300 mg at bedtime resulted in a response in 90% of patients (8). However, adverse reactions to lithium are common and require monitoring of blood levels. Furthermore, titration to adequate plasma concentrations is difficult, and many patients discontinue treatment owing to side effects (24, 31). Indomethacin is an effective prophylactic agent for HH. Prophylactic doses of indomethacin between 25 and 150 mg/day have shown favorable results in approximately 70% of the patients with HH (32). There was an absolute response to indomethacin by day 3 at a dose of 25 mg, three times daily (23). It has been hypothesized that HH is associated with changes in cerebrospinal fluid (CSF) pressure and that indomethacin exerts its therapeutic effect by regulating the CSF pressure (33, 34). The patient declined the above three treatment options owing to concerns about coffee-induced insomnia, lithium-related adverse effects, and aggravation of ulcers by indomethacin.

The orexins are two neuropeptides that are derived from prepro-orexin via proteolytic cleavage. They are synthesized in the lateral, posterior, and periventricular hypothalamic nuclei (35). The hypothalamic orexinergic system may be a crucial pathway in circadian rhythm disorders like narcolepsy, cluster headache, and HH. Daridorexant, a recently developed dual orexin receptor antagonist, proved advantageous in improving wake after sleep onset and latency to persistent sleep in elderly individuals with insomnia disorder (36). The recent randomized, double-blind, placebo-controlled phase 3 trials further confirmed the safety and efficacy of daridorexant in insomnia disorder patients (37). However, there are currently no reported cases of using orexins for prophylactic treatment of HH.

Melatonin (MT) is considered beneficial for circadian rhythm disorders and may be beneficial for HH treatment (8, 10, 20, 23, 24, 38). It is the only prophylactic drug reported for the treatment of HH in children (39). Melatonin is an indole heterocyclic compound with a short half-life (≤20 min). It is produced only at night by the pineal gland, and its biological clock properties are mediated by two G protein-coupled melatonin receptors associated with different signaling mechanisms in the SCN, including driving the sleep–wake cycle (40). MT1 receptors inhibit the SCN and induce sleep, whereas MT2 receptors induce changes in SCN signaling that convert the sleep–wake cycle into a light–dark cycle. Melatonin receptors resynchronize irregular circadian rhythms and are beneficial for sleep architectures (41). 5-hydroxytryptamine (5-HT)2C receptors are the only serotonin receptors involved in circadian rhythms. 5-HT2C receptors agonists can simulate the effects of light on the SCN and suppress the production of melatonin (42). They can inhibit melatonin release by mediating light information in the SCN during early night via a post-synaptic mechanism (43–45). Therefore, both 5-HT and melatonin can regulate circadian rhythmicity. Ramelteon is a selective MT1/MT2 agonist approved by the Food and Drug Administration as a hypnotic. Ramelteon has an elimination half-life of approximately 1–2 h, which is much longer than that of melatonin (38). Ramelteon has been reported to yield good results in a patient with HH. This suggests that ramelteon is a potential prophylactic agent for HH (38). However, melatonin and ramelteon are not available as prescription medications in Mainland China. Agomelatine possesses both melatonergic agonist and complementary 5-HT2C antagonist properties (46, 47). It has a stronger affinity for MT1 and MT2 receptors and a longer half-life than melatonin (48). The effects of agomelatine on sleep–wake rhythms in rats have also been studied (49). Agomelatin administration before dark exposure enhanced REM and slow-wave sleep in rats. The decrease in wakefulness induced by agomelatine was different from that induced by melatonin and ramelteon, which may reflect an interaction between melatonin and the 5-HT2C receptor. These findings provide a rationale for HH treatment using agomelatine.

## Conclusion

In the real world, HH only occurs during sleep, leading to substantial sleep disturbances in older populations. Headache center neurologists need to focus on the prophylactic treatment of patients before bedtime to avoid nocturnal awakenings. Agomelatine may be a potential prophylactic treatment option for HH, particularly in patients with caffeine-induced insomnia, lithium-related adverse effects, or a history of ulcers.

## Data availability statement

The original contributions presented in the study are included in the article/supplementary material, further inquiries can be directed to the corresponding author.

## Ethics statement

The studies involving human participants were reviewed and approved by Ethics Committee of the First Hospital of Shanxi Medical University. Written informed consent was obtained from the patient for the publication of this case report.

## Author contributions

LL, W-xS, and J-yS conducted the study. S-yX and LL wrote the first draft. S-yX and C-xL conceptualized the study and revised the manuscript. All authors contributed to the article and approved the submitted version.

## Funding

This study was supported by grants from Doctoral Fund of the First Hospital of Shanxi Medical University (YB161706, BS03201631, and SD2215); Shanxi Applied Basic Research Program (201801D221426, and 20210302124404).

## Conflict of interest

The authors declare that the research was conducted in the absence of any commercial or financial relationships that could be construed as a potential conflict of interest.

## Publisher’s note

All claims expressed in this article are solely those of the authors and do not necessarily represent those of their affiliated organizations, or those of the publisher, the editors and the reviewers. Any product that may be evaluated in this article, or claim that may be made by its manufacturer, is not guaranteed or endorsed by the publisher.

## Abbreviations

5-HT: 5-hydroxytryptamine
CSF: Cerebrospinal fluid
HH: Hypnic headache
ICHD-3: International Classification of Headache Disorders, 3rd edition
MT: Melatonin
MRI: Magnetic resonance imaging
OSAS: Obstructive sleep apnea syndrome
REM: Rapid eye movement
SCN: Suprachiasmatic nucleus
VBM: Voxel-based morphometric
